# Voltage-dependent potassium channel regulatory subunits in the immune system

**DOI:** 10.1007/s12551-026-01412-3

**Published:** 2026-02-11

**Authors:** Magalí Colomer-Molera, Silvia Cassinelli, María Navarro-Pérez, Antonio Felipe

**Affiliations:** Molecular Physiology Laboratory, Departament de Bioquímica I Biomedicina Molecular, Institut de Biomedicina (IBUB), Universitat de Barcelona, Avda. Diagonal 643, 08028 Barcelona, Spain

**Keywords:** Ion channels, Regulatory subunits, Immune system, Lymphocytes, Antigen-presenting cells

## Abstract

The immune system depends on ion channels to control activation and maintain cellular homeostasis. The role of voltage-dependent potassium channels (Kv) in immune cells has been well studied in recent decades, with a special interest in the role of Kv1.3 in cell physiology and its implications in autoimmune diseases. However, native K^+^ currents in leukocytes result not only from the assembly of pore-forming α-subunits but are also shaped by regulatory β-subunits that fine-tune gating, trafficking, and pharmacology. Immune cells express members of the Kvβ, KCNE, and KChIP families, but the contribution of these regulatory subunits to immune physiology remains largely underexplored. In this review, we synthesize evidence for regulatory subunit expression and function in leukocytes, focusing on how these partners modify Kv channel behavior and downstream signaling. We highlight Kv1.3-Kvβ2.1-KCNE4 as a promising immunoregulatory complex, and we discuss the role of KChIPs in shaping gene expression as well as a Kv regulatory subunit. Despite gaps in the expression of regulatory subunits in immune cells, increasing evidence highlights the importance of further studies addressing the role of Kvβ-subunits in the immune context. Understanding how Kv channels are regulated in leukocytes could lead to new ways to control immune responses and develop new targeted therapies.

## Introduction

The immune system comprises a highly coordinated network of cells that respond to internal and external threats. The immune response relies on two main systems: innate and adaptive immunity (Chaplin 2010). The innate immune system provides immediate defense through cells such as macrophages, neutrophils, dendritic cells, and natural killer cells, which recognize common features of pathogens. The adaptive immune system, which is composed of T and B lymphocytes, develops specific responses against individual antigens. Both systems interact closely, with innate cells activating adaptive responses and adaptive cells enhancing innate defenses (Wang et al. 2024).

Immune cells rely on calcium (Ca^2+^) as a second messenger to regulate functions such as gene expression, proliferation, apoptosis, and migration (Cahalan And Chandy 2009). Ca^2+^ influx following cell activation modulates the activity of enzymes and transcription factors that ultimately lead to immune system activation. Intracellular oscillation of Ca^2+^ modulates the membrane potential. To maintain a proper electrochemical gradient, immune cells efflux potassium (K^+^), allowing the necessary hyperpolarized membrane potential to sustain Ca^2+^ entry (Feske et al. 2015). K^+^ channels, including voltage-gated potassium (Kv) channels, are the proteins that mediate the K^+^ efflux necessary for Ca^2+^ influx. Besides Kv channels, the calcium-activated KCa3.1 channel is also crucial for maintaining the membrane potential in T and B lymphocytes (Cahalan And Chandy 2009; Grissmer et al. 1993). Kv and KCa channel expression in T lymphocytes changes depending on the differentiation state (Beeton et al. 2006; Ghanshani et al. 2000; Wulff et al. 2003).

While the role of Kv channels in the immune system has been largely explored, less is known about the role of the Kv regulatory subunits in this physiological context. In this review, we offer a summary of the regulatory proteins that affect Kv channel function and integrate this knowledge in an immune system framework. Because there is limited data in Kv channel regulatory subunit expression and function in immune cell systems, we have also incorporated relevant in vitro data that should be considered in this context.

## Voltage-gated potassium channels in the immune system

Kv channels are pore-forming tetrameric protein complexes that allow selective K^+^ efflux in response to membrane depolarization. By controlling membrane potential, they regulate excitability, calcium signaling, and cellular activation (Armstrong And Hille 1998). In the immune system, the most relevant Kv channels are Kv1.3 and Kv1.5, which shape activation, proliferation, and cytokine secretion, making them key modulators of immune responses and potential therapeutic targets (Beeton et al. 2006; Navarro-Perez et al. 2024).

Kv1.3 is essential for the activation and proliferation of leukocytes. Upon antigen presentation by antigen-presenting cells, T-cell receptor signaling activates PLCγ, leading to calcium release from the endoplasmic reticulum (ER). This depletion triggers calcium entry through calcium release-activated channels (CRAC) formed by the association of STIM1 and Orai1 proteins. In addition to Kv1.3, the calcium-activated intermediate-conductance potassium ion channel KCa3.1 also participates in generating the membrane potential to sustain this calcium influx, which is crucial for downstream signaling. An increase in intracellular calcium activates the phosphatase calcineurin, which in turn drives the nuclear translocation of nuclear factor of activated T cells (NFAT) and promotes the transcription of activation-related genes (Cahalan And Chandy 2009). Kv1.3 expression is relatively low in naïve and central memory T cells, whereas higher levels are detected in activated effector memory T cells, which are abundant in chronic inflammation and autoimmune disorders. Kv1.3 has been postulated to be a therapeutic target for autoimmune diseases such as multiple sclerosis, psoriasis, and rheumatoid arthritis in animal models (Navarro-Perez et al. 2024). Most pharmacological efforts have focused on blocking Kv1.3 in T cells to treat autoimmune diseases.

Kv1.3 is also expressed in macrophages (Vicente et al. 2006), B cells (Szabo et al. 2015), and other cell types (Bergermann et al. 2019; Du et al. 2017; Szabo et al. 2015). In macrophages, Kv1.3 is necessary to promote apoptosis (Leanza et al. 2012), a process that has been linked to the association of Kv1.3 with Cav1 and its expression in mitochondria (Capera et al. 2021). Kv1.5 is also expressed in macrophages, where it participates in phagocytosis and migration processes (Park et al. 2006). In antigen-presenting cells, Kv1.3 and Kv1.5 are found in heteromeric complexes (Vicente et al. 2006), where the subunit composition determines the cellular output (Kotecha And Schlichter 1999).

Kv1.1 is also expressed in human helper CD4⁺ T cells (Freedman et al. 1995) and plays an anti-inflammatory role. Blocking Kv1.1, either with DTX-K or selective anti-Kv1.1 antibodies, triggers TNFα production (Fellerhoff-Losch et al. 2016). Kv1.1 appears to counteract the activity of Kv1.3, which promotes T-cell activation (Beeton et al. 2006; Wulff et al. 2003). However, KTX, a Kv1.1 and Kv1.3 blocker (Giangiacomo et al. 2004), has an immunosuppressive effect, suggesting that the role of Kv1.3 in T lymphocytes is greater than that of Kv1.1 (Beeton et al. 2001).

## Kv channel regulatory subunits

The functional diversity of Kv channels is increased by the expression of regulatory subunits that diversify and fine-tune their function. These regulatory subunits influence channel gating kinetics, membrane trafficking, and pharmacological sensitivity, adapting Kv signaling to specific cellular contexts. The main protein families include the Kvβ, KCNE, KChIP, and DPP families, as well as various scaffold and adaptor partners. While the role of regulatory subunits has been well established in excitable tissues, such as Kv1-Kvβ and Kv7.1-KCNE1 in cardiomyocytes (Barhanin et al. 1996) and Kv4-KChIP-DPP interactions in the brain (Marionneau et al. 2009), much less is known about how these subunits contribute to Kv channel function in immune cells and their implications for the pharmacological targeting of Kv channels. In this review, we summarize the state of the art of Kvβ, KCNE, KChIP, and DPP proteins and their role in the immune system. 

### Kvβ

**Fig. 1:**
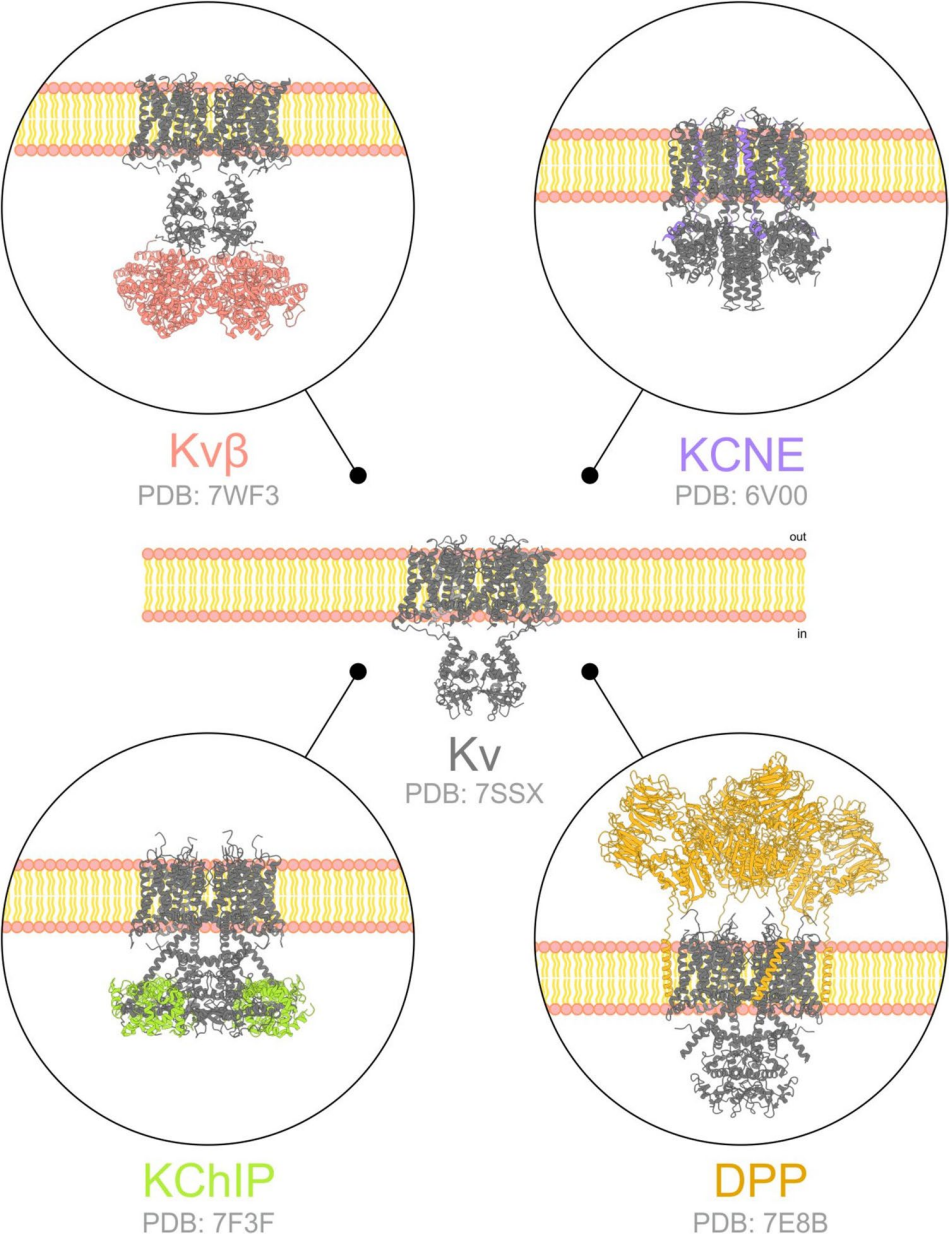
Voltage-gated potassium channel regulatory subunits. Kv channels are regulated by members of several ancillary families. The protein structure has been deciphered and is indicated by the protein database (PDB) reference number. The Kv channel is depicted in gray. The regulatory subunits are indicated in pink (Kvβ), violet (KCNE), green (KChIP), and orange (DPP). The membrane topology is also indicated to orient the protein complex

Kvβ subunits were the first discovered family of Kv auxiliary proteins to interact with and modulate the activity of α-subunits (Fig. 1) (Pongs et al. 1999; Scott et al. 1994). In mammals, three Kvβ genes produce regulatory subunits, namely, Kvβ1.1–1.3.1.3, Kvβ2.1–2.2.1.2, and Kvβ3.1 (Heinemann et al. 1995; Schultz et al. 1996). Structurally, Kvβ have a variable 70–90 amino acid N-terminal and an ~330 residue-long conserved core (Campomanes et al. 2002). Some Kvβ proteins induce rapid N-type inactivation in Kv channels by blocking the ion-conducting pore with the variable Kvβ N-terminal (Bahring et al. 2001). In the Kvβ2 structure, the core domain is a triosephosphate isomerase barrel with a bound NADP^+^ cofactor, conferring redox sensitivity to the regulatory subunit (Gulbis et al. 1999; Weng et al. 2006).

The Kv1.2–Kvβ2 crystal structure confirmed a 4:4 stoichiometry and revealed details of the interactions between the α-subunits and β-subunits (Long et al. 2005). Several residues from the tetramerization domain of the Kv channel constitute the docking surface for the Kvβ protein that assembles with the pore-forming subunits as a preformed tetramer. The four Kvβ subunits face the cytoplasm, allowing the flow of ions through lateral negatively charged openings above the T1–Kvβ complex, which become occluded by N-type inactivating peptides (Gulbis et al. 2000). The Kv–Kvβ interaction enlarges the cytoplasmic portion of the channel, providing a larger surface for the binding of other regulatory proteins, such as kinases or phosphatases (Kwak et al. 1999; Wang et al. 2004; Williams et al. 2002), as well as a link to the cytoskeleton (Nakahira et al. 1998; Proepper et al. 2014).

Kv1 channels in physiological contexts are usually associated with Kvβ2 (Monaghan et al. 2001; Rhodes et al. 1997; Shamotienko et al. 1997). Kvβ2 does not induce N-type inactivation and instead increases α-subunit oligomerization stability (Shi et al. 1996). Kv-Kvβ interaction starts early during channel biogenesis in the endoplasmic reticulum and promotes surface expression for some channels (Ma et al. 2001; Nagaya And Papazian 1997; Shi et al. 1996). Increased plasma membrane channel abundance is observed in parallel to increased potassium current amplitude (Accili et al. 1997; Campomanes et al. 2002).

Lymphocytes induce the expression of Kvβ subunits in response to different proinflammatory stimuli, such as IL-2 (Autieri et al. 1997). Kvβ2.1 accumulates in plasma membrane lipid raft microdomains in the immunological synapses of T lymphocytes during the immune response (Roig et al. 2022). Kvβ2.1 is a Kv1.3 regulatory subunit responsible for increasing the current amplitude of the channel (McCormack et al. 1999; Tyagi et al. 2022). Kv1.3 is also located in the immunological synapse, where it modulates the calcium response after T-cell receptor engagement (Nicolaou et al. 2009; Panyi et al. 2004a; Rus et al. 2005). Both Kvβ1 and Kvβ2 are expressed in macrophages (Vicente et al. 2005), and Kv1.3 and Kv1.5 are the main voltage-dependent α-subunits in these cells (Vicente et al. 2006). In this context, Kvβ1.3 induces Kv1.5 N-type inactivation (Decher et al. 2008) and alters its pharmacology (Gonzalez et al. 2010). In addition, the Kvβ1.2 subunit is responsible for PKC-dependent phosphorylation of the channel (Fischer et al. 2014; Williams et al. 2002).

### KCNE

**Fig. 2:**
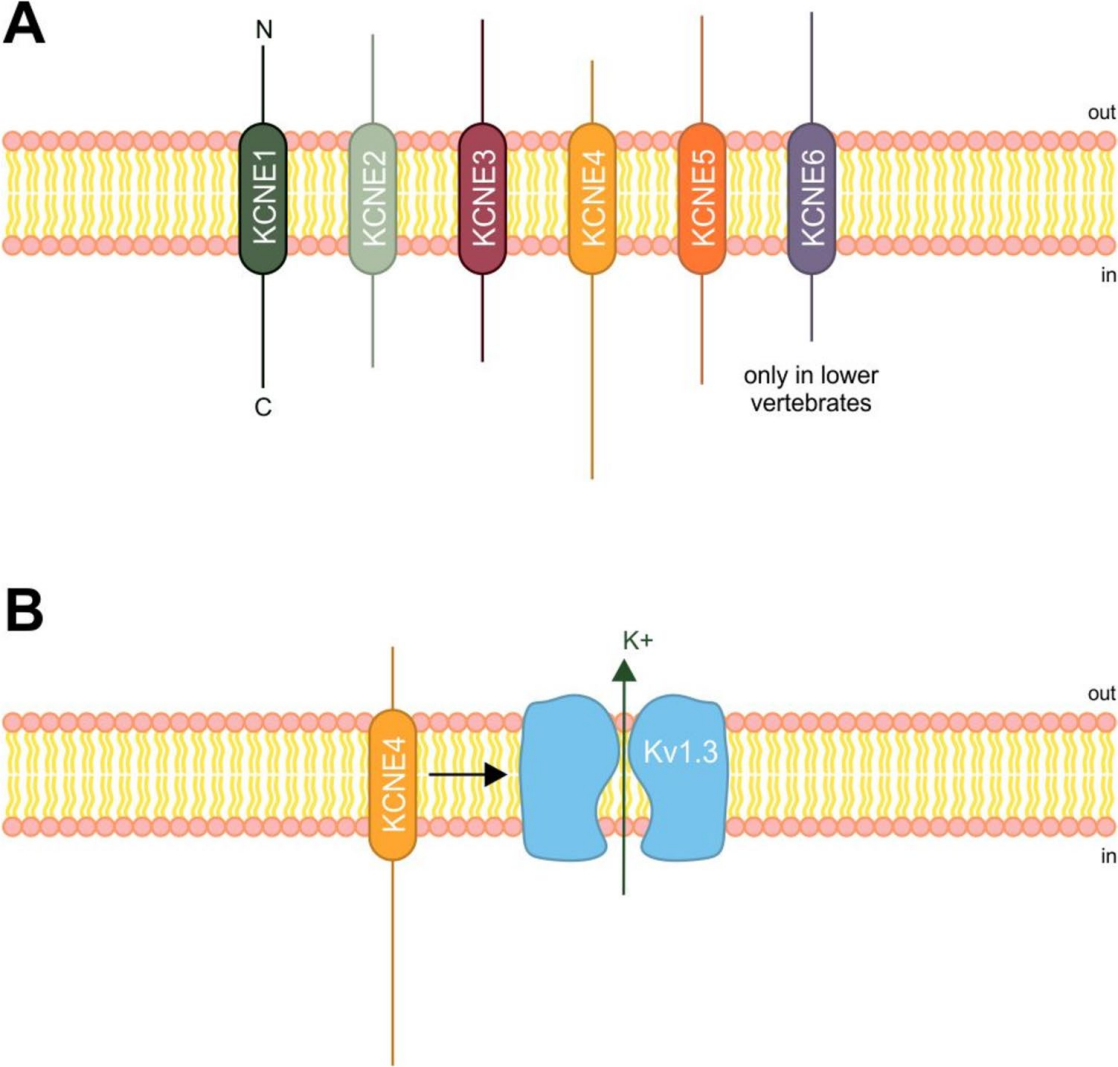
The KCNE family.** A** Members of the regulatory-subunit KCNE family. KCNE1-6 proteins have been identified. Although not in scale relative to the transmembrane domain, the relative lengths of the N- and C-terminal domains of the protein are indicated. **B** KCNE4 is a Kv1.3 regulatory subunit. KCNE4 interacts with the Kv1.3 channel via its C-terminal and transmembrane domains

The founding member of the KCNE family (KCNE1) was discovered by injecting fractionated rat kidney RNA into *Xenopus oocytes* to induce potassium ion channel-like currents (Takumi et al. 1988). It was first named minimal K^+^ channel protein (MinK), and subsequent family members discovered by sequence homology were named MinK-related peptides (MiRP1-4) (McCrossan and Abbott 2004). MinK and MiRP1-4 were later renamed potassium voltage-gated channel subfamily E (KCNE1-5). A sixth KCNE member (KCNE6) has been identified as a functional regulatory subunit of Kv channels in lower vertebrates (Fig. 2A) (Kasuya et al. 2024). KCNE proteins have limited sequence homology but share a single-pass transmembrane topology with a flanking extracellular N-terminal that can undergo glycosylation and an intrinsically disordered cytoplasmic C-terminal domain (Malaby And Kobertz 2013). The structure of the transmembrane domains of KCNE1 and KCNE3 has been solved via nuclear magnetic resonance and recognized as part of a Kv7.1 complex by cryo-electron microscopy, respectively (Kang et al. 2008; Sun And MacKinnon 2020). For other KCNE members, the length of the transmembrane alpha helix has been determined through circular dichroism spectroscopic measurements (Bates et al. 2025; Campbell et al. 2022; Coey et al. 2011).

KCNE proteins associate promiscuously with Kv channels from different families, including Kv1 (Fig. 2B) (Grunnet et al. 2003; Kanda et al. 2011), Kv2 (David et al. 2015), Kv3 (Abbott et al. 2001; McCrossan et al. 2003), Kv4 (Abbott 2016; Wang et al. 2014; Wu et al. 2010), Kv7 (Roura-Ferrer et al. 2009; Sanguinetti et al. 1996; Sun And MacKinnon 2020), and Kv11 (Anantharam et al. 2003; Um And McDonald 2007). Kv–KCNE regulatory outcomes include changes in single-channel conductance, gating kinetics, modulation of voltage dependence, and alteration of the subcellular localization of the complex.

**Fig. 3:**
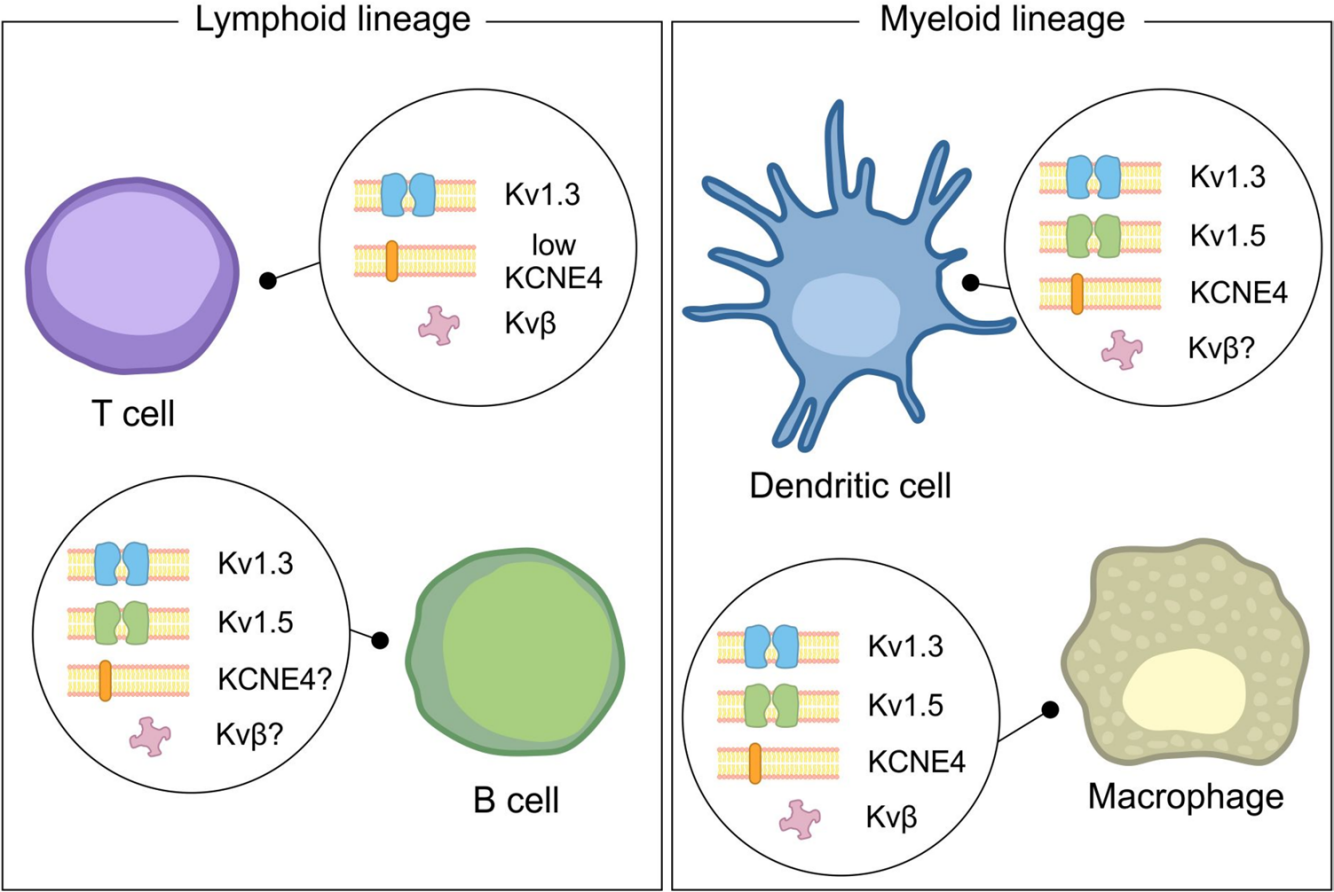
Immune system cells express Kv and various regulatory subunits. Cells from both the lymphoid and myeloid lineages express a limited repertoire of Kv and some regulatory partners. Although the mRNA expression of all Kv partners has been detected in leukocytes, the presence of protein is, in some cases, debatable or has not yet been analyzed (Sole And Felipe 2009; Sole et al. 2013)

The roles of KCNE subunits in the immune system have been the subject of only a limited number of studies. KCNE1 is expressed in macrophages (Cheng et al. 2013) and in T lymphocytes (Attali et al. 1993; Ben-Efraim et al. 1994; Chabannes et al. 2001), but no clear role has been defined for this regulatory subunit in leukocytes. KCNE3 in tumor-associated macrophages has been linked to anti-inflammatory M2 macrophage polarization, suggesting the usefulness of blocking KCNE3 for improving cancer therapeutics (Liu et al. 2025). KCNE4 is abundantly expressed in antigen-presenting cells, including macrophages (Sole et al. 2009; Vicente et al. 2005) and dendritic cells (Mullen et al. 2006; Vallejo-Gracia et al. 2021), and has been detected in lower quantities in T lymphocytes (Fig. 3) (Vallejo-Gracia et al. 2021). KCNE4 is a potent regulator of the T lymphocyte Kv1.3 channel (Wulff et al. 2003), which is also present in antigen-presenting cells alongside Kv1.5 (Kotecha And Schlichter 1999; Pannasch et al. 2006; Vicente et al. 2006; Villalonga et al. 2007). KCNE4 is responsible for the inhibition of Kv1.3 currents (Grunnet et al. 2003) via ER retention of the channel (Sole et al. 2009) through a C-terminal juxtamembrane tetraleucine motif (Sole et al. 2019). This ER retention is also promoted by the transfer of a potent ER-related retention motif of KCNE4 to the oligomeric complex (Roig et al. 2021). In addition, the transmembrane domain, together with the C-termin, enhances the C-type inactivation of the channel (Sastre et al. 2024b) in a stoichiometry-independent fashion (Sole et al. 2020). KCNE4 dimers also compete for binding with Kv1.3 and calmodulin (Roig et al. 2021). In contrast, KCNE4 does not regulate Kv1.5 function (Grunnet et al. 2003; Sole et al. 2016), although this issue is the subject of debate (Crump et al. 2016).

### KChIP

Kv channel-interacting proteins (KChIPs) are specific Kv4 regulatory subunits with calcium-binding properties that belong to the neuronal calcium sensor family (An et al. 2000) (Fig. 1). The KChIP family includes four members (KChIP1-4) with four EF-hand-like calcium-binding motifs in their conserved C-terminal domain (Morohashi et al. 2002; Ren et al. 2003) and a variable N-terminal domain responsible for the distinct modulation of Kv4 channels (Jerng And Pfaffinger 2008). Alternative splicing of the four KChIP genes produces a diverse set of proteins that further increase Kv4 channel function (Baranauskas 2004). Isoforms from the same gene can have completely opposite effects on the same channel (Decher et al. 2004). KChIPs participate in Kv channel assembly at the endoplasmic reticulum level and forward plasma membrane trafficking (Foeger et al. 2010). Kv–KChIP complexes maintain a 4:4 stoichiometry (Kim et al. 2004a; Kunjilwar et al. 2013) where KChIP subunits can heteroassemble (Zhou et al. 2015) in the cytoplasmic domain of the α-subunits, anchored in a lateral position of the T1 domains (Kim et al. 2004b; Wang et al. 2007). Kv4–KChIP complexes are further regulated by the presence of DPP subunits (Kise et al. 2021).

Kv4 channels are responsible for transient A-type currents (*I*_*SA*_) in neurons and cardiac transient outward currents (*I*_*to*_) in cardiomyocytes (Maffie And Rudy 2008; Nerbonne And Kass 2005; Serodio et al. 1996). In this context, KChIP1-4 are expressed in the brain (Marionneau et al. 2009), and only KChIP2 is present in the heart, where it interacts with Kv4.2 and Kv4.3 (Niwa et al. 2008; Rosati et al. 2001). Although Kv4 channels have not been reported to be expressed in immune cell lineages, KChIPs are expressed in some immune cell types. KChIP2 is expressed in T and B lymphocytes, where it inhibits the expression of IL-2, IL-4, and IFN-γ (Savignac et al. 2005). B lymphocytes also express the voltage-gated Kv1.5 channel (Vallejo-Gracia et al. 2013), and KChIP2 impairs Kv1.5 plasma membrane trafficking in HEK293 cells (Li et al. 2005). The KChIP3 isoform KChIP3.1, also known as downstream regulatory element antagonistic modulator (DREAM) (Carrion et al. 1999), is expressed in mature B lymphocytes, where it reduces the levels of IgM and IgG antibodies (Savignac et al. 2010). In T lymphocytes, KChIP3 participates in regulating proliferation and reducing proinflammatory IL-2, IL-4, and IFN-γ cytokine production (Savignac et al. 2005), acting as an immune response repressor. Overall, the role of KChIPs in the immune system seems unrelated to Kv channel activity and dependent on their role as Ca^2+^-dependent transcription factors (Mellstrom et al. 2014).

### DPP

Dipeptidyl peptidases (DPPs) are single-pass transmembrane serine proteases with an N-terminal cytoplasmic domain and a C-terminal extracellular domain that undergoes glycosylation (Fig. 1). Among them, DPP-like (DPPL) members DPP6 and DPP10 lack enzymatic activity because the catalytic serine residue is replaced in DPP6 and DPP10 by an aspartic acid (Asp712) and a glycine (Gly651), respectively (Bezerra et al. 2015; Chen et al. 2006; Kin et al. 2001; Qi et al. 2003). DPPLs associate with Kv4 complexes (Jerng et al. 2005, 2004; Radicke et al. 2005; Zagha et al. 2005) in a 4:4 stoichiometry for DPP6 (Soh And Goldstein 2008) and a variable stoichiometry with a preference for the 4:2 (Kv4:DPP) configuration for DPP10 (Kitazawa et al. 2015). The structure of Kv4.2–DPP6 reveals an octameric complex with four subunits from each protein as well as a dodecameric architecture with an additional four KChIP subunits (Kise et al. 2021). DPPLs regulate Kv4 activity by increasing unitary single-channel conductance and promoting plasma membrane trafficking of the channel, increasing the current amplitude of Kv4 (Kaulin et al. 2009; Lin et al. 2014; Zagha et al. 2005)*.* Kv4 inactivation and recovery from inactivation are also accelerated by DPPLs (Jerng et al. 2007, 2004; Radicke et al. 2005; Takimoto et al. 2006).

DPP6 is a Kv4 regulatory subunit responsible for A-type K currents in CA1 hippocampal neurons (Sun et al. 2011). DPP6 accelerates S4 movement in Kv4.2 (Dougherty And Covarrubias 2006). DPP6 is also found in resting mast cells, monocytes, and activated dendritic cells present in the immune microenvironment of uterine leiomyosarcoma (Ke et al. 2022). DPP10 is expressed in the hippocampus, neocortex, cerebellum, and main olfactory bulb, where it forms ternary complexes with Kv4 and KChIPs (Wang et al. 2015). DPP10 was found in CD4 memory resting T cells in ulcerative colitis (Xue et al. 2021) and in eosinophils, neutrophils, and T and B lymphocytes in patients with asthma (Sim et al. 2022).

Although both DPP6 and DPP10 are present in some immune cells or are related to immune function, the lack of information regarding Kv4 expression in immune cells suggests a Kv-independent mode of action. It seems that DPP10-expressing cells participate in ERK phosphorylation, leading to inflammation and remodeling in NSAID-exacerbated respiratory disease patients (Sim et al. 2022). To further understand the role of DPPLs in the immune system, further research is needed.

## The role of scaffold proteins in immune system Kv channel function

Scaffold proteins participate in the formation of complexes and binding of proteins to specific locations in the cell and are relevant for amplifying signal transduction (Shaw And Filbert 2009). Structurally, they contain globular domains linked by unstructured regions with crucial binding functions (Balazs et al. 2009). Although scaffold proteins are not considered a class of Kv regulatory subunits, they participate in shaping Kv channel function in several contexts, including immune system physiology.

Members of the membrane-associated guanylate kinase (MAGUK) family, such as disc large 1 (Dlg1, also known as synapse-associated protein SAP97), are scaffold proteins first cloned in humans in B lymphocytes (Lue et al. 1994), which tightly regulate Kv1.3 expression, location, and function at the plasma membrane in dendritic cells (Dong et al. 2019; Hanada et al. 1997). In T lymphocytes, SAP97 promotes Kv1.3 binding to the lymphocyte-specific protein tyrosine kinase Lck (Kuras et al. 2012; Zhou et al. 2015), contributing to lymphocyte activation (Xavier et al. 2004). In Kv1.5, SAP97 interacts with the channel through the N-terminal domain (Eldstrom et al. 2003; Folco et al. 2004; Murata et al. 2001) and increases its activity through plasma membrane anchoring (Abi-Char et al. 2008; Mathur et al. 2006). In dendritic cells, SAP97 maintains Kv channel expression, and dendritic cell-specific knockouts reduce the antibody response capacity as a consequence of decreased Kv activity (Dong et al. 2019).

Disc large 4 (Dlg4), also known as PSD95 (postsynaptic density protein 95) or SAP90, also interacts with the C-terminal PDZ domain of Kv1.3 channels in T lymphocytes, promoting the polarization and colocalization of Kv1.3 and CD4 in the immunological synapse (Doczi et al. 2011; Hajdu et al. 2015; Szilagyi et al. 2013).

## Kv1.3, Kvβ2.1, and KCNE4 in the immune system

The literature about the Kv regulatory subunits in the immune system is mainly focused on Kvβ2.1 and KCNE4. Therefore, we next discuss the role of these two subunits in the immune system as an emerging hypothesis, providing a systematic synthesis and immune-focused integration on the current literature. Kvβ2.1 and KCNE4 are two Kv1.3-interacting partners expressed in the immune system, where Kv1.3 activity mediates cell activation in cells from both lymphoid and myeloid lineages (Beeton et al. 2006; Di Lucente et al. 2018; Nguyen et al. 2017). Kvβ2.1 enhances Kv1.3 currents via plasma membrane targeting in human lymphocytes (McCormack et al. 1999), whereas KCNE4 has the opposite effect and reduces plasma membrane abundance of the channel in vitro (Sole et al. 2009).

Kvβ subunits are expressed in T lymphocytes (Fig. 3) and are upregulated during mitogen-dependent activation (McCormack et al. 1999). Kv1.3 also participates in proliferation, and channel blocking in lymphocytes inhibits cell mitogenesis (Panyi et al. 2004b). In contrast, KCNE4 overexpression in Jurkat T lymphocytes inhibits Kv1.3 activity and decreases cell activation and proliferation (Vallejo-Gracia et al. 2021). Additionally, in T cells, KCNE4 expression is altered in a cell cycle-dependent manner and sharply increases in response to PMA/PHA activation (Sole et al. 2013). During the immune response, Kv1.3 is localized at the immunological synapse between T lymphocytes and antigen-presenting cells (Capera et al. 2024; Nicolaou et al. 2009; Panyi et al. 2004a). Kvβ2.1, during TCR engagement and subsequent activation, is also concentrated at the immunological synapse within lipid raft microdomains (Beeton et al. 2006; Roig et al. 2022).

In macrophages, lipopolysaccharide (LPS)-dependent activation leads to the upregulation of both Kv1.3 and KCNE4, whereas treatment with dexamethasone downregulates Kv1.3 without affecting KCNE4 expression (Sole et al. 2009). Murine macrophages also express the Kvβ1 and Kvβ2 subunits (Vicente et al. 2006). Kvβ2.1 expression is also increased in response to LPS and macrophage colony-stimulating factor (M-CSF) (Vicente et al. 2005); however, Kvβ2.1 expression remains constant upon activation in humans (McCormack et al. 1999). Kv1.3 currents in macrophages exhibit different inactivation kinetics depending on the cell activation stimulus (Vicente et al. 2005), most likely as a consequence of differential Kv1.3 regulatory subunit expression. KCNE4-dependent regulation of Kv1.3 includes a marked increase in C-type inactivation of the channel in vitro (Sastre et al. 2024b).

The putative role of KCNE4 as a Kv1.3 modulator in the immune system is also interesting from a therapeutic perspective. The C-terminal KCNE4 polymorphism rs12621643 is linked to immune-related diseases such as childhood acute lymphoblastic leukemia (Trevino et al. 2009) and allergic rhinitis (Freidin et al. 2013). Kv1.3 has been studied as an interesting pharmacological target for autoimmune disease treatment (Navarro-Perez et al. 2024), and KCNE4 has been shown to affect Kv1.3 blocking by intracellular small molecules in vitro (Sastre et al. 2024a), suggesting the importance of KCNE4 in Kv1.3 pharmacology.

## Future perspectives

Despite significant progress in understanding the role of Kv channels in the immune system, many questions remain open regarding the contribution of their regulatory subunits. The main limitations of the current literature can be categorized in (i) expression profiling, (ii) in vivo stoichiometry, (iii) novel interactions, (iv) disease implication and pharmacology, and (v) broader physiological context.

### Expression profiling

There is a lack of information on systematic differential protein expression. Several advances have been made, but mostly focus on transcriptomic analysis (Autieri et al. 1997; Sole et al. 2013). To better understand Kv regulation, exhaustive proteomic profiles of β subunit expression in different immune cell types and under different activating stimuli are needed.

### In vivo stoichiometry

Once exhaustive protein expression profiles have been determined, we need to take into account the physiological subunit composition of the channel complexes. Most of the information currently available at the protein level focuses on two Kv1.3 regulatory subunits, namely, Kvβ2.1 and KCNE4. Most of the current data is focused on individual subunit–channel interactions and based on the results of in vitro experiments. In addition to expanding in vivo knowledge, studying the possibility of a Kv1.3–Kvβ2.1–KCNE4 complex and how it affects Kv1.3 function would be interesting. Depending on the cell type model, we could further explore a Kv1.3–Kv1.5–Kvβ2.1–KCNE4 channelosome with different subunit stoichiometries.

### Novel interactions

The diversity of Kv regulatory subunits leaves room to explore new Kv-β subunit interactions in the context of the immune system. KChIPs play important roles in immune cell control, but they have only been described as transcription factors that negatively impact the production of proinflammatory cytokines (Carrion et al. 1999; Mellstrom et al. 2014; Savignac et al. 2005), not as Kv channel regulators. However, both KChIP2 and Kv1.5 are expressed in B lymphocytes (Savignac et al. 2005; Vallejo-Gracia et al. 2013), and KChIP2 affects Kv1.5 plasma membrane trafficking in vitro (Li et al. 2005). Studying the possibility of this interaction in a physiological system could help further expand our current knowledge.

### Disease implication and pharmacology

Once the importance of regulatory Kv subunits has been established, we need to carefully examine the role of mutations in the β subunits that could affect their function. Numerous mutations in members of the KCNE family impact their ability to properly regulate Kv channels (Abbott And Goldstein 2002). In KCNE4, a distal C-terminal polymorphism, rs12621643, is correlated with immune-related diseases such as childhood acute lymphoblastic leukemia (Trevino et al. 2009) and allergic rhinitis (Freidin et al. 2013). Pharmacological approaches targeting Kv channels should also consider the presence of regulatory subunits (Sastre et al. 2024a) and explore how targeting regulatory subunits may allow for more extensive immunomodulation than blocking Kv channels alone.

### Broader physiological context

Finally, Kv channels are only a subset of the ion channels expressed in immune cells, and their function shapes—and is shaped—by overall ion flux. For a comprehensive understanding of immune cell physiology, knowledge of different ion channel families needs to be integrated. The β subunits presented in this review can also participate in the regulation of non-Kv channels, such as K_Ca_ channels (Chen et al. 2024; Khanna et al. 1999; Levy et al. 2008), further extending their possible roles in shaping the immune response.

## Conclusions

Voltage-gated potassium channels, particularly Kv1.3 and Kv1.5, are key regulators of immune cell function and activation. Among their auxiliary partners, Kvβ subunits have emerged as important modulators of Kv channel function within the immune system, especially in partnership with Kv1.3 in T lymphocytes. KChIPs also play relevant roles as transcription factors in lymphocytes, inhibiting proinflammatory cytokine production, although their potential role as Kv1.5 channel inhibitors in immune contexts remains largely unexplored. Scaffold proteins such as SAP90 and SAP97 are also involved in lymphocyte physiology via Kv1.3 interactions. KCNE4, another Kv1.3 regulatory subunit, is also present in immune cells and plays a promising role as an immune modulator. Overall, further understanding these complex regulatory networks is essential for identifying new immunomodulatory mechanisms and therapeutic opportunities.

## Data Availability

No datasets were generated or analyzed during the current study.
